# Workplace-based learning opportunities in a South African family medicine training programme

**DOI:** 10.4102/phcfm.v15i1.4073

**Published:** 2023-10-09

**Authors:** Neetha J. Erumeda, Louis S. Jenkins, Ann Z. George

**Affiliations:** 1 Department of Family Medicine and Primary Care, Faculty of Health Sciences, University of the Witwatersrand, Johannesburg, South Africa; 2 Gauteng Department of Health, Ekurhuleni District Health Services, Germiston, South Africa; 3 Division of Family Medicine and Primary Care, Faculty of Medicine and Health Sciences, Stellenbosch University, Cape Town, South Africa; 4 Western Cape Department of Health, George Hospital, George, South Africa; 5 Department of Family, Community and Emergency care, Faculty of Health Sciences, University of Cape Town, Cape Town, South Africa; 6 Centre of Health Science Education, Faculty of Health Sciences, University of the Witwatersrand, Johannesburg, South Africa

**Keywords:** decentralised clinical training, family medicine, family physician, postgraduate training, postgraduate registrars, workplace-based learning, workplace learning opportunities

## Abstract

**Background:**

Workplace-based learning (WBL) provides authentic learning opportunities to develop fit-for-practice healthcare workers. Different types of WBL opportunities have been described in high-income countries, but the opportunities in the district health systems of sub-Saharan Africa have not been characterised.

**Aim:**

This study explored family physicians’ (FPs) and registrars’ perceptions of WBL opportunities in a decentralised postgraduate family medicine registrar training programme.

**Setting:**

The study was conducted at five decentralised training sites across two provinces affiliated with the University of the Witwatersrand in South Africa.

**Methods:**

This instrumental case study involved semi-structured qualitative interviews with 11 FPs and 11 registrars purposively sampled across the training sites. The verbatim transcripts were thematically analysed using Braun and Clark’s six-step approach.

**Results:**

Workplace-based learning opportunities were grouped into four themes: Learning from interpersonal interactions, learning from district activities, self-directed learning and contextual influences on learning opportunities. Registrars learnt from patients, peers, FPs and other professionals. Feedback, self-reflection, portfolio use, involvement in various district events, such as student and staff teaching, and continuous medical education augmented learning. Contextual influences originated from health facilities, resource availability, district management and university support, excessive workload and a need for standardised district learning opportunities.

**Conclusion:**

Registrars are exposed to several types of WBL opportunities in district health systems. Effective engagement with available opportunities and addressing contextual challenges could enhance registrar learning.

**Contribution:**

Maximising learning opportunities to promote registrars’ acquisition of required skills and competencies to efficiently address community needs in a middle-income country such as South Africa.

## Introduction

The World Health Organization’s (WHO) Astana declaration (2018) advocated strengthening primary health care (PHC) services and prioritising universal health coverage, focusing on preventive, promotive, curative and rehabilitative care services.^1^ The training of skilled health workers to address community needs is a global priority.^1,2^ Medical training institutions continually modify curricula to equip postgraduate trainees with the knowledge, skills and professional attitudes needed to address community needs.^2,3,4,5,6,7^ Workplace-based learning (WBL) involves trainees learning in the workplace by meeting the clinical service delivery requirements and utilising authentic contexts for optimal training and skills development.^8^ Studies on WBL have been conducted in high-income countries such as the Netherlands and the United Kingdom, but very few studies have been conducted in sub-Saharan Africa, including South Africa (SA), on workplace learning experiences. An in-depth exploration of views of WBL opportunities in postgraduate training based in an SA district health context is still lacking. This article describes the various types of WBL opportunities and workplace factors influencing WBL in the district health context of SA.

## Learning in the workplace

Workplace-based learning ranges from learner-centred, informal, unstructured learning in the absence of a trainer to formal learning in the presence of a mentor or supervisor.^8^ Informal WBL lacks the defined curriculum, timetables and linear teaching that characterise formal teaching at the university level, resulting in criticisms about WBL being disorganised and opportunistic.^8^ Despite a perceived lack of educational rigour, process and structure, WBL is becoming the preferred strategy.

Workplace-based learning draws on several learning theories, including cognitive, socio-cognitive and social constructivist theories.^8^ The cognitive theories emphasise individuals learning from their own experiences and self-reflective processes, while the socio-cognitive and social-constructivist theories focus on learning from social interactions.^8^ In cognitive theories, learning is seen to happen during the interplay between existing and new knowledge.^9^ In socio-constructive theories, learning is visualised as part of everyday practice and occurs by engaging with peers, supervisors and other healthcare team members. Informal learning at the workplace involves implicit, reactive and deliberative learning.^10^ Implicit learning refers to knowledge acquisition that happens with no conscious attempt to learn; reactive learning is about spontaneous intentional learning that occurs while performing an action, and deliberative learning involves planning towards defined learning goals.^10^

Self-regulated and self-directed learning are integral components of WBL. In self-regulated learning, trainers regulate learning primarily in academic environments. In contrast, self-directed learning involves trainees developing their learning goals, identifying activities and resources and seeking external feedback at their workplaces.^11^ While self-directed learning is moderated by internal processes such as self-reflection, it is also modified by external educational interventions, such as feedback, assessments and learning portfolios.^12^ Self-directed learning consists of short loops that are triggered during consultations with a lack of knowledge during minor learning activities at the work place.^12^ Long loops include more extended learning periods based on complex problems, such as difficulties in communication or handling cases such as child abuse.^12^

## Factors affecting workplace-based learning

Several personal, interpersonal and contextual factors affect WBL. Personal factors that enhance learning include learner behaviours such as active involvement, accountability, professionalism, conscientiousness and acceptance of criticism.^13^ Prior experience, knowledge, motivation, attitude and confidence also affect trainees’ learning.^14,15,16^ A lack of concentration, difficulties in dealing with negative feedback and managing work and private life, and being passive learners with a lack of motivation act as barriers.^12,13,17^ Trainees’ WBL is enhanced when using workplace artefacts such as case reports, reflective logs or portfolios.^16^

Interpersonal factors affecting WBL arise from trainees’ interactions with peers, supervisors, other professionals and patients.^16,18,19,20^ More WBL takes place during interactions with peers and supervisors^18^ and as part of informal discussion or handovers than when trainees work alone.^19^ Peer interactions motivated trainees to put in extra effort to reach their peers’ standards.^19^ Interaction with a supervisor affirms trainees’ behaviours, actions and decision-making during patient consultations and helps them apply their knowledge.^21^ Other motivational factors are supervisor, mentor or peer engagement and feedback offered in a safe environment,^15^ good supervisor-trainee relationships, supervisory commitment, opportunities for supervision, and mutual observation and dialogue.^13^ In contrast, poor supervision and supervisory relationships hinder learning.^12^

Inter-professional or intra-professional interactions promote informal and formal learning^16,18,22^ through reflective practice, spontaneous or triggered, implicit through participation, increasing self-awareness and developing coping mechanisms.^16^ Learning is enhanced by social integration, various task allocations, and successes and mistakes.^23^ Trainees also learn from engagements with patients^20^ and their families, through reflecting on patient encounters, especially with difficult or peculiar patients,^22^ critical incidents and patient communication.^24^ During patient interactions, trainees identify deficits in medical competencies but not always their lack of general competencies, such as communication skills and ethics.^19^

Workplace-based learning needs a supportive clinical environment, as it directly influences patient-care practices.^25^ A conducive WBL environment requires material resources, an appropriate patient mix and clinician-to-patient ratio, sufficient clinical trainers, a manageable workload, protected time for learning and support from relevant stakeholders.^26,27^ Creating a positive learning environment with sufficient trainee support and knowledgeable, passionate and skilled trainers is vital.^26,27,28^ A collaborative learning climate encourages reflection, feedback, debriefing, supervision and guidance from supervisors.^14^ Organisational factors, such as the layout of the work environment, interpersonal dynamics among team members and the availability of complex patients^14^ augment learning.^16^ Health system factors such as high supervisor and trainee workloads and high patient volumes prevent trainee observations.^13^

## Workplace-based learning in South Africa

Two SA studies identified several factors that promoted and hindered WBL experiences. One study of postgraduate registrars (SA trainees) in a laboratory setting categorised the origin of these factors as the university, workplace, home circumstances and personal.^29^ Academic role models and supportive trainers who enjoyed teaching promoted learning, while unstructured academic activities, feelings of demotivation, conflicting family and work responsibilities and negative supervisor feedback hindered learning.^29^ A family medicine (FM) study identified context, adequate utilisation of a learning portfolio, patient consultations, engagement with clinically relevant supervisors and providing sufficient feedback as promoting learning.^30^

Registrar learning in workplaces in post-graduate FM decentralised training at the University of the Witwatersrand (Wits University) in SA is primarily based on self-directed and self-regulated learning. The university curriculum and national learning outcomes, as determined by the SA Academy of Family Physicians (FPs), are readily accessible in the public domain.^6,31^ Registrars develop individualised learning plans aligned with the national learning outcomes, according to their learning goals in workplace settings. The activities they undertake to achieve their goals, including setting timelines and finding resources, are augmented by guidance from their supervisors.^31^ Workplace-based learning involves clinical and educational supervision by FPs or specialists in various disciplines during clinical rotations, the annual compilation of a learning portfolio and formative and summative assessments.

The study reported in this article forms part of a broader mixed methods case study, evaluating a postgraduate FM decentralised training programme by using a complex programme evaluation logic model. The previously published articles from the larger study reported on resource availability, postgraduate supervision and supervisory feedback evaluated as the inputs, processes and outputs of the logic model.^32,33,34^ This article reports on the FPs’ and registrars’ perceptions of types of the learning opportunities, registrars’ learning behaviours and the learning environments of the decentralised postgraduate FM training.

## Research methods and design

### Study design

Case studies explore, in-depth, the multiple perspectives of the complexity and uniqueness of a phenomenon in a ‘real life context’ of a ‘bounded system’, such as a programme or an event^35^ by using various data sources and data-collection methods.^36^ An instrumental case study focuses on a particular case to gain in-depth insight into an issue or to redraw a generalisation.^35^ An instrumental case study investigating the various aspects of the ‘phenomenon of interest’, that is, the postgraduate FM decentralised registrar training programme, in the context of Wits University was conducted.

### Study setting

This qualitative study was conducted at five decentralised training districts affiliated with Wits University. There were four study sites (Ekurhuleni, Johannesburg Metro, Sedibeng and West Rand) in Gauteng province and a fifth site in the Dr Kenneth Kaunda district in the North West province.

Wits University registrar training involves 3 years of district-based training, including annual 3-weekly training blocks at the university and a fourth year of elective rotations. District training takes place across various facilities, including community health centres, which are 24-h facilities providing primary healthcare services, run primarily by nurses^37^ with support from general practitioners and FPs. The smaller district hospitals provide comprehensive health services and are staffed mainly by general practitioners, with a few specialists, including FPs.^38^ Regional hospitals support district hospitals and are led by resident specialists.^37^ Regional training centres are based in community health centres or district hospitals and are the sites for weekly academic engagements between FPs and the registrars. Registrars rotate for 2–3 months in various clinical departments of the district or regional hospitals, depending on the training district. Registrars typically engage in academic discussions or presentations on various clinical or non-clinical topics, facilitated by FPs in groups or one-on-one sessions. Family physicians facilitate registrars’ case presentations at community health centres or district hospitals and supervise the registrars according to the learning portfolio requirements. Specialists and senior doctors in various disciplines supervise the registrar learning during clinical rotations.

### Study population and sampling

The target population comprised 20 FPs and 21 registrars. These individuals were purposively sampled^39^ because they were perfectly positioned to provide in-depth insights into the WBL opportunities in the programme. Purposive sampling involves the deliberate selection of individuals or sites^39^ to achieve representativeness across settings, capture adequate homogeneity of the study population by obtaining a range of variations and examining critical cases to compare or illuminate the differences between settings or individuals.^40^ The FPs and registrars had varying exposures to and experiences of WBL opportunities in the decentralised training programme depending on their roles and responsibilities in each district. We included FPs and registrars from all five training sites for broader geographical representation.

All second-and third-year registrars in the programme were invited to participate. First-year registrars were omitted as they did not have adequate training experience to contribute meaningfully. Registrars in their fourth year and beyond were excluded as they were completing their elective rotations and were not actively involved in the yearly training programme. Family physicians, who are jointly appointed by the university and the relevant provincial health departments, were invited to participate. Recently qualified FPs who had not registered as specialists and were not joint appointees were excluded. All participants who were invited from the FPs and registrars agreed to participate in the study. The final samples consisted of 11 FPs and 11 registrars.

### Data collection

The principal author conducted 80-to-90-min semi-structured interviews between March and August 2020. Eight face-to-face interviews were conducted with the remainder (*n* = 14) on Zoom or Microsoft Teams because of the coronavirus disease 2019 (COVID-19) lockdown. Table 1 represents the interview guide. The interview process continued until data saturation was reached.^41^ The interviews were audio-recorded, transcribed verbatim and checked for fidelity. The registrars and FPs transcripts were open-coded^42^ (first level of coding into individual segments) separately. Codes were compared and contrasted across the transcripts for each participant group. No new codes were identified after nine interviews from each group, but two more were interviewed from each group to ensure data saturation.

**TABLE 1:** Interview guide.

Registrar questionnaire	Family physician questionnaire
How would you describe your learning environment in your district?	How would you describe the learning environment for registrars in your district?
What are the learning opportunities for you in the district?	What are the learning opportunities for the registrars in your district?
What do you think about your learning behaviours in your district?	What do you think about the learning behaviours of your registrars in your district?
What do you think about the opportunities for interprofessional learning in your district?	What do you think about the opportunities for interprofessional learning in your district?
What do you think about your use of portfolio as a learning tool?	What do you think about the use of portfolio as a learning tool by your registrars?
What do you think about the feedback given to you by the family physician?	What do you think about the feedback given to your registrar by you?
How do you reflect on the feedback given to you by the family physician?	What do you think about the registrar reflection on the feedback given to them by you?
What do you think are the challenges for your learning in the district?	What do you think are the challenges for registrar learning at your district?

FP, family physicians.

### Data analysis

Braun and Clark’s six-step approach to thematic analysis was used to analyse the transcripts.^41^ The steps are: (1) familiarisation with the data; (2) generation of initial codes; (3) searching for themes; (4) reviewing themes; (5) defining and naming themes; and (6) producing a final report.^41^ MAXQDA 2020 (Verbi Software, Berlin, Germany) was used to manage the analysis.

The initial coding system was checked by the two co-authors until agreement was reached on the naming of codes. The final coding system was applied to transcripts through an iterative process of coding and checking until agreement was reached about grouping codes into categories and themes, and reviewing and naming the themes.

### Trustworthiness

Frequent discussions between the authors improved the intercoder reliability and the credibility of the findings.^43^ A codebook was developed and constantly revised until the coding system was finalised, further improving credibility.^44^ Detailed descriptions of the study methods, including setting, inclusion and exclusion criteria, and data collection, augmented the transferability.^45^ Data triangulation from the two groups of participants also improved credibility and dependability. Dependability of the findings were further improved by recording decisions made from the emergent data at a particular time and their rationale, and how various codes and categories were grouped, themes developed, reviewed^46^ and renamed during analysis.

Reflexivity relates to the degree of bias that the researcher intentionally or unintentionally introduces into the research.^47^ The primary author was cognisant that her position as a supervisor and colleague to some participants may have influenced their responses. She mitigated these by reassuring them about the anonymity and confidentiality of the findings. She also self-critiqued and constantly reflected on her personal biases and assumptions in a journal to improve the confirmability. The co-authors were not involved in the postgraduate FM training being evaluated. Their reflexivity centred around their roles as medical educators – they reflected, examined and explored their interpretations of the findings based on their involvement and experience in postgraduate FM training.

### Ethical considerations

Ethical clearance to conduct this study was obtained from the University of the Witwatersrand Human Research Ethics Committee (Medical) (No. M191140). Permission to conduct the study was obtained from five districts through the National Health Research Database (GP_201910_050). Informed written consent was voluntarily obtained from study participants.

## Results

The age of the registrar participants’ (RG1–RG11) ranged from less than 30 to 60 years. Six registrars were in their third year and five were in their second year of study. Registrars had PHC experience before joining as community service doctors, medical officers or in private practice. The FP participants’ (FP1–FP11) ages ranged from 41 to 60 years and most had more than 5 years of training experience (see Table 2).

**TABLE 2:** Participants’ characteristics.

Characteristics	Registrars’	Family physicians’
**Age**		
≤ 30	3	-
31–40	6	-
41–50	1	8
51–60	1	3
**Sex**		
Male	5	7
Female	6	4
**Nationality**		
South Africa	7	5
Not South Africa	4	6
**Year of training**		
Year 3	6	-
Year 2	5	-
**Years of training experience**		
1–5 years	3	-
> 5 years	8	-

**FIGURE 1:**
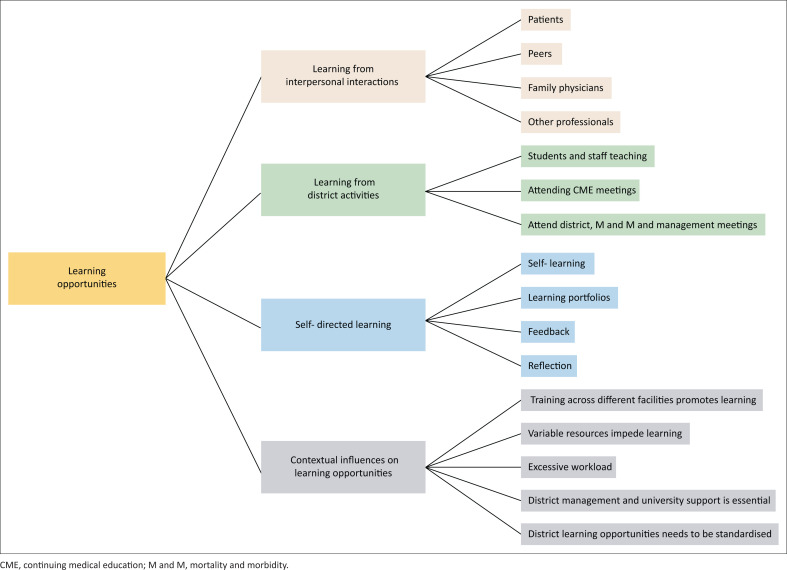
Themes and subthemes.

The four themes identified were classified according to the learning opportunities from people, activities, self and the environment. Figure 1 shows the sub-themes within each theme.

### Learning from interpersonal interactions

Registrars perceived that the learning opportunities were primarily from patient interactions, other professionals and FPs. The FPs agreed with registrars but thought there were peer learning opportunities for registrars during training.

#### Patients

Registrars and FPs felt that the registrars had adequate opportunities to learn from undifferentiated and complex patients with various conditions. These challenges broadened their thinking processes:

‘You get the opportunity to be exposed to so many available, let me say, good patients that will come with many challenges at once. So, a complex patient. You’ll have this very often that challenges you as a clinician to broaden your thinking and to do.’ (RG 6)

Most registrars recognised the importance of taking each patient encounter as a learning opportunity. Each patient motivated them to apply FM principles, read the literature and critique their approach to patient management:

‘I’m having to at least take one patient a day, go back home, read the way that my supervisor has shown me, look at what case I had, and look at whatever … identify at least one problem where the patient … look at the literature … and also critique my approach to the patient.’ (RG 10)

#### Peers

Family physicians thought registrars learn by interacting with senior registrars:

‘People like RG 3 [*registrar*] were senior at a point in time. When they were junior[*s*], there were people who were senior[*s*] ahead of them. Now RG 4 and them are consultants; they are still around. RG 3 are consultants, [*and*] they are still around. So now we have RG 6 and RG 1. So, there are opportunities for intergenerational learning.’ (FP 9)

#### Family physicians

Registrars mentioned discussing patients with FPs at their workplaces as an excellent learning opportunity. During patient consultations, the presence of FPs provided opportunities for registrars to identify their strengths and challenges, which motivated them to read more about the patient:

‘I have access to my supervisor, even outside our formal working time. So, I’m free to discuss cases that I encounter with him, so this gives me an opportunity to learn. I can go back and search in terms of resources on the case that I encountered … I did discuss it with him, and then with my supervisor, who will also give his input, so, from this is [*sic*] a very good learning opportunity.’ (RG5)

Family physicians agreed with registrars that they had more opportunities to practice FM principles in the presence of their supervisors:

‘Where the registrar is placed, and he or she can make use of all those tools adequately and necessary, with supervision or with help, is important.’ (FP 10)

In contrast, many registrars felt they did not have adequate opportunities to observe role modelling, either because of the lack of availability of FPs but more often because of the small number of FPs or their multiple roles. Registrars wanted to observe more of their consultant’s approach to patients and their families, which was sometimes challenging:

‘Just … sufficient family physicians … I think it would add a lot of value if we had … if we worked with family physicians, it would help us to learn how to be a family physician. So how to approach things, what is their viewpoint and how to more readily interact with the community and family aspect of the patients [*sic*].’ (RG 7)

#### Other professionals

Both registrars and FPs described how they learned from other professionals, including nurses, physiotherapists and others who worked with registrars at clinics and hospitals. Participants had mixed opinions on learning from other professions at the clinic level and most registrars agreed that more opportunities arose in the hospitals rather than at clinics:

‘Definitely at the bigger hospitals, I think it’s good, and I have attended like some of their training, especially with the physiotherapists, in say, in orthopaedics, and OT [*occupational therapy*], with the making of different splints and exercises.’ (RG 8)

‘The opportunities are abundant, I think, in that the nursing staff or other sections of the clinics within the district are always open and willing to [*answer*] any questions that you have or if you want to know how their systems work, or … get them to teach you a skill, then those opportunities are always available.’ (RG 7)

Family physicians raised concerns that despite various opportunities to learn from other professions, registrars did not maximally utilise them, even at the clinic level:

‘I tell this registrar, you’ve got an optometrist who’s got a big machine that we’ve just bought for her, why are you struggling to know how to do fundoscopy … You can make a time and go to her and say, I’m going to come to you every Friday for one hour for the next two months. So, the opportunities are there, but I’m not so sure whether our registrars are using it on their own.’ (FP 9)

Some registrars agreed with FPs that they could have made better use of interprofessional learning opportunities:

‘I think our learning is quite fragmented. You mostly learn from the doctors, from the family physicians. We do sometimes get involved in student teaching. But in terms of your multidisciplinary level of teaching involving nurses, physiotherapists, OT [*occupational therapists*] … it’s from person-to-person interest, but there’s not much integrated learning.’ (RG 1)

### Learning from district activities

According to FPs and registrars, registrars’ active involvement in training medical and other students and staff is an essential component of WBL learning. Most registrars and FPs concurred that registrars had opportunities to participate in district CME (Continuing Medical Education) activities, community-oriented primary care with home visits and mortality and morbidity meetings, which enhanced their learning.

#### Students and staff training

Registrars and FPs felt that registrars training junior doctors, supervising interns or students, and conducting student assessments provided registrars good learning opportunities:

‘By giving an opportunity to [*the*] registrar to train other, for example, giving in-service training, training junior doctors, training interns, by giving them [*the*] responsibility to supervise, also that’s going to help them.’ (FP 2)

Some registrars perceived that utilising those opportunities augmented their ability to gauge individual knowledge levels and structure training sessions according to the level of various student groups:

‘There is something to learn in terms of teaching styles, how to conduct a teaching or training session, how to gauge the level of knowledge in terms of how you should focus your training for the different levels of students. Firstly, you won’t treat the final year the same as a third-year or a clinical associate, first-year as a fifth-year medical student. So, you need to be able to gauge the content need(s) of the students.’ (RG 1)

#### Attending CME meetings

According to registrars and FPs, another learning opportunity was participating in CME meetings and workshops where different professionals presented and interacted with registrars:

‘Like there’s a couple [*of training courses*] that I actually attended last year that I thought there is something happening. I have attended something to do with malnutrition, I have attended mental health [*training*], I have attended HIV [*human immunodeficiency virus course*], so I think, yes, when there is something going on, they do inform us, and they do allow us to go.’ (RG 9)

#### Attend district, mortality and morbidity and management activities

Family physicians mentioned that participating in district community-oriented primary care and quality improvement projects facilitated registrar learning at the workplace:

‘And also apart from the clinical care, then his [*the registrar’s*] research, QIPs [*quality improvement project*], the COPCs [*community-oriented primary care*] with the WBOT [*ward-based outreach team*]. I want him [*registrar*] to be involved.’ (FP 10)

Another critical opportunity was the registrars’ participation in the mortality and morbidity meetings, where the group discussed missed opportunities in patient management and registrars learned how to improve patient care in similar situations:

‘There’s M and Ms [*mortality and morbidity*] meetings that I’ve gotten to attend at PHC 3, I think that’s been a learning opportunity where you sit, you see what mistakes have been made, and even before you make that mistake, you’re now in a better position [*because*] you’ve learnt from the case. So, I think M and Ms are also a good opportunity … to also, as a professional, give feedback on what could have been done.’ (RG 2)

A significant concern raised by a few FPs was that registrars were insufficiently exposed to leadership roles to prepare them to take on these roles once qualified as specialists:

‘Especially maybe our senior registrars, looking back in terms of even when we were registrars, say, for example, we never attended any senior meeting. Now suddenly you are qualified, and suddenly you must attend senior meetings, you must make decisions, but you’ve never been technically trained in terms of…’ (FP 1)

### Self-directed learning

Family physicians and registrars thought that learning occurred during registrars’ self-directed learning activities such as self-learning, reflection, feedback and compiling their learning portfolio. Both groups of participants varied in their opinions on registrar learning behaviours, approach to feedback, reflection and utilisation of a learning portfolio.

#### Self-learning

Some FPs felt that some registrars did well on self-directed learning, with exceptional efforts to keep up to date with current knowledge. In contrast, other registrars failed to understand the self-directed learning process and struggled to manage it:

‘So, it’s not something that you can generalise. So, it’s actually varied. There are some who are independent; they do their work, and quality work and they come, and then you really see that this person knows what they’re doing. But then there are some who are just completely off; they just don’t have a clue what they’re doing, and they are problematic.’ (FP 11)

One FP commented that registrars believed they knew what adult education was when they did not:

‘A lot of them assume they know what (the) adult education model is. But I don’t think they do. I think they feel adult education is about staying back and doing everything on their own, and that’s not it.’ (FP 9)

On registrar learning behaviours, FPs in some districts felt registrars displayed variable behaviours:

‘There is always one or two who struggle, and it’s likely we know because we easily identify them and work on it, but often it’s not successful.’ (FP 6)

In contrast to this, others thought that registrars were committed and improved their behaviour:

‘It’s exceptional. They’re committed to learning. I’m impressed with their learning behaviour, and I’m impressed with their dedication. They have really improved tremendously, and they are willing to learn as well.’ (FP 4)

The FPs in some districts commented on the lack of professionalism and work ethic, but opinions varied among FPs:

‘So, there are good people [*who*] really go the extra mile. And then there are other registrars that have [*a*] poor work ethic and complain a lot, and don’t get the work done, so we’re definitely working with different personalities.’ (FP 7)

In contrast, all the registrars believed they had good learning behaviours and work ethic, were willing to learn, and participated in various registrar activities:

‘I think I’m always willing to learn, and I’m always willing to be part of what needs to be done, and if there’s training, I’m always looking forward to it and I’m also contributing if needed to, and I’m not being pushed.’ (RG 9)

#### Learning portfolios

All registrars and FPs agreed that a learning portfolio is an exceptional tool to support self-directed learning, providing ample guidance on learning objectives and how to achieve them:

‘So it serves as a guide to me, and it serves also as a check book [*sic*] where I can always go back and see which objectives or learning plans I have set, and if I’m still on track with those learning plans, and if I’ve managed to achieve those objectives, and if I’m satisfied with the achievement that I have, or if I’m lagging behind and I might not achieve what I set for myself as a goal.’ (RG 6)

Despite being identified as a good learning tool, most registrars and FPs agreed that adequate utilisation of the portfolio by registrars was problematic. Often, portfolios were regarded as last-minute paper-based exercises when they had to be submitted and were not used to stimulate deeper learning:

‘It’s not something that we are putting emphasis on or sort of…we don’t really use that to guide us. We sort of more keep note of what the deadlines are and try to keep up to them. So, it’s more a deadline-driven process, I would think, rather than sort of using it as a material to guide us.’ (RG 7)

#### Feedback

Registrars felt that they incorporated feedback, whether positive or negative. They felt encouraged by positive feedback and viewed negative feedback as a learning opportunity to address deficiencies:

‘So, it depends on whether it’s positive or negative feedback. So, if it’s negative feedback, that means I’m going to have to go [*and*] read more, or to improve in whatever the remark or the feedback was [*about*]. And if it’s positive feedback, you feel encouraged.’ (RG 4)

Some registrars disclosed that they struggled to incorporate feedback when they started the programme:

‘I was actually very frustrated by some of the feedback when I first started because there was a lot of negative feedbacks and… [*laughs*] and left me frustrated. I remember going to one of my fellow registrars and, oh, I want to cry, I feel frustrated in the programme, because everything I get is negative; it’s almost like I don’t do anything right.’ (RG 3)

Some FPs thought most registrars could engage with feedback more and act accordingly. They said that registrars kept repeating the same mistakes, implying that they failed to understand the feedback:

‘Sometimes, to be very honest to you, not 100% effective. Many of them still struggle with incorporating the feedback with regards to improving, that’s the thing, that’s what I can tell you. Because I see that, sometimes I say, but I told you this last time and I gave you this feedback and all that.’ (FP 3)

#### Reflection

Family physicians believed that more reflection on their patients and the feedback they received could provide better opportunities for self-directed learning, instead of the variable reflection they thought was being undertaken:

‘The reflection is something that is also a little bit neither here nor there. Because if you see…you give a registrar feedback and then when he comes next time and the same problem crops up, then you begin to realise actually this registrar did not reflect on the feedback that you give.’ (FP11)

In contrast, registrars believed that they reflected on supervisor feedback, especially on the strengths and challenges addressed, and they attempted to improve themselves in future patient encounters:

‘But we have to reflect on what feedback we’re given in terms of our strengths, our weaknesses, what we’re doing well, what we’re not doing well, and then you reflect on that and try and improve your skills around the weaknesses that you are facing.’ (RG 1)

### Contextual influences on learning opportunities

Registrars and FPs agreed that the learning environment was conducive to learning, with adequate patient numbers and various levels of hospitals, but the resources were variable. Contextual challenges included excessive workload, a lack of adequate support from district management and the university, and a lack of standardisation, negatively influencing registrar learning.

#### Training across different facilities promotes learning

Both participant groups agreed that training across various district health facilities, including PHC clinics and district and regional hospitals, allowed access to patients with different sociodemographic characteristics and high burden of disease, which added to the richness of the learning environment:

‘We have the district hospital and then you have the PHCs, and then we have the regional hospital. So, when we are at [*sic*] the district we’re able at least as registrars in family medicine, we’re able to see cases that are sometimes then referred from the regional hospital, and it gives you an idea what is for the district hospital, which cases are supposed to go to.’ (RG4)

‘We’re in a district where more than 45% of adults are unemployed. So, the social determinants of health, they are [*visible*] here; if there’s any place where they impact, it’s here. So, the burden of disease, the environment, is good for learning.’ (FP 9)

One registrar commented that she got better learning opportunities in smaller hospitals with a stronger primarycare focus, compared with bigger hospitals:

‘I wouldn’t maybe learnt as much as I’ve learnt in these three years, by not given the opportunity and being put into the position, in such small facilities where you have to learn to, number one, make do with what you have, and you have to teach yourself and you need to make sure that you are up to date.’ (RG 8)

#### Variable resources impede learning

Family physicians agreed that FP numbers had improved during the years in some districts, which was encouraging, but there was still a challenge in other districts. Both participant groups commented on the variability of available human and material resources across the districts:

‘I think in terms of the skills, like clinical, especially like OSCE skills, that sometimes is a little bit challenging because there’s sometimes a lack of resources. You know, for example, there’s no like speculums to do like a pap smear, or things like that.’ (RG10)

‘I think has improved over the years with regards … They have the materials, the mannequins, the books, the opportunities to learn with tools around in the clinics … everything is actually provided for them; it’s just a question of the trainers.’ (FP 3)

#### Excessive workload

Registrars and FPs expressed challenges of heavy workloads as part of service delivery, negatively impacting learning and requesting service delivery support from their workplace:

‘Sometimes there are so much patient load [*sic*] at the clinic that most of the times we find ourselves doing service delivery more than learning. Then because there would be a shortage of staff, we also do clinical care, so that’s a challenge.’ (RG 5)

‘Obviously, we want to develop registrars or specialists with tenacity. But where they are pushing queues, it’s also a problem.’ (FP 9)

#### District management and university support is essential

According to the FPs, registrars need support from the district management to promote learning. They wanted protected time for registrar learning and allowing clinical rotations at hospitals:

‘They [*district management*] don’t like this idea of registrar going outside for a clinical rotation, because they feel we’re reducing the staff that provide clinical service at primary health care [*clinics*]. So, there’s been this on-going rift that the district is paying the registrars but they’re working in the hospital. And they tend not to understand that this is a training programme.’ (FP 4)

Family physicians requested university engagement to create awareness about FM training in the district, which could augment learning opportunities:

‘I think Wits [*University*] needs to come in here and assist us so that if they can talk at a higher level and say, look, the registrars are at training, much as they are part of the workforce but they need to be … people need to understand that their quality of work, or whatever they are doing, needs to be linked with their objective, which is learning.’ (FP 11)

Meanwhile, registrars appreciated accessibility to university resources such as the library:

‘I think that we have access to the resources and references via the Wits Health Sciences library, and so whatever I do, I can always go back and review the best standard practice.’ (RG7)

#### District learning opportunities need to be standardised

Some registrars and FPs emphasised that registrars’ learning in the districts was variable, and there was a need to standardise learning opportunities:

‘I’d love a structured approach, or even, can I say, a global structure. Almost to know what are other districts doing? Like are we behind, are we forward? Where are we in terms of everyone else? Like a universal plan … like I know you can’t, you can’t universally make every district do the same thing, but it would be nice to know what the other districts are doing to see relative to what are we doing.’ (RG 11)

## Discussion

This study identified multiple types of learning opportunities for registrars, including interactions with various groups of people, participation in district activities and self-learning strategies. However, the WBL opportunities available were used variably both within and across districts. Compared with previous studies from high-income countries, this study in a middle-income country contributes a different perspective on what is needed to enhance WBL in authentic clinical settings of PHC.

Regular interactions with complex and undifferentiated patients in authentic clinical settings provided WBL opportunities. Patient engagements and reflections from those interactions are considered among the best WBL strategies for trainees.^12,16,19,23,27^ Registrars learned by integrating the FM principles, reading about patients’ conditions and developing self-directed learning behaviours. Patient interactions also helped registrars to practice more soft skills, including communication skills, clinical reasoning and professional behaviour.

Registrars had adequate opportunities to learn from peers and FPs in most districts. Peer learning interactions and sharing views and experiences among trainees in different years are recognised WBL strategies.^27^ Interestingly, peer learning opportunities identified only by the supervisors underscored the importance of intergenerational learning, which was not recognised by registrars. Family physicians thought registrars were more pressurised to learn when they identified knowledge gaps in the presence of peers, as found elsewhere.^19^ Previous studies perceived that learning from peers was more acceptable for registrars than from supervisors, as supervisor presence could interfere with a safe learning environment.^19^ In contrast, interactions with FPs were identified as excellent opportunities for registrar reflections on their strengths and weaknesses. Family physician accessibility during and after work hours encouraged registrars to integrate the FM principles or tools, positively influencing WBL. Interactions with peers and supervisors provide excellent learning opportunities^12,18,19,48^ and constitute examples of situated learning.^49^ Role modelling could potentially demonstrate excellent clinical teacher, human and professional behaviours^50^ and has a greater impact on trainees to internalise those behaviours than any other teaching method.^51^ A lack of role-modelling opportunities hindered WBL in this study, as identified elsewhere.^20^ Peer teaching^48^ and supporting trainers are also essential to optimise WBL opportunities in clinical settings.^3^

Participants identified learning from other types of professionals as essential for WBL. Previous studies in high-income countries showed that learning occurs between health professionals during workplace interactions.^18,19,20,21,22,23^ Interprofessional learning between various health professionals working in collaboration is a WHO recommended strategy to address health systems challenges worldwide.^52^ According to most participants, although interprofessional learning was a good learning opportunity, it was insufficiently utilised. Trainees were often reluctant to spend time learning from other professions, perhaps because they felt it was not part of their assessments. Other reasons could be the non-recognition of others’ expertise, professional stereotypes and hierarchical challenges.^16,53^ Workplace-based learning opportunities from other professions varied across clinics and district hospitals, and registrars identified more opportunities at the district hospital. For example, dietary advice offered by dieticians could be observed and utilised by the registrars for managing a diabetic or hypertensive patient. Similarly, basic or advanced counselling skills learnt from a psychologist could be applied to patients with mental-health illnesses while practising holistic care. A recent SA study reiterated the importance of trainees’ learning from other professions, as it improves collaboration and teamwork, professional satisfaction and patient care.^54^

The availability of a range of other learners has been found to enhance learning opportunities.^26,51^ The participants in this study reported that registrar training of students and staff enhanced learning by providing them with a platform to gain practical experience towards becoming an effective trainer, an expected FP role in SA.^55,56^

In WBL, self-directed learning, reflection, and the ability to incorporate internal feedback at expected standards and external feedback from peers or supervisors^15^ are vital for trainee personal and professional development.^16,57^ Reflection is essential to learning from experience^57^ and it develops registrars as lifelong learners.^58^ Incorporating feedback, reflection and self-directed learning skills were variable among the registrars in this study, as perceived by FPs, but registrars thought they integrated feedback sufficiently into their learning. The ownership for self-directed learning, looking actively for learning opportunities, reflection and feedback, improves during training because of trainees’ increased awareness.^28^ The senior registrars in our study commented more than juniors on their reflection-on-action, on patient interactions after their daily work, looking into the supervisors’ feedback and addressing knowledge gaps. Despite these processes being only learnt during training, FPs expected registrars to engage with feedback and reflection from the time they joined the programme, as found previously.^28^ Instead, supervisors could provide more guidance to registrars with various WBL strategies earlier in their training. The participants agreed that workplace artefacts such as learning portfolios, case logs and reflective logs were excellent WBL tools, as seen in other studies.^16,30^ The portfolio was only used as a last-minute paper-based exercise for yearly submission but was not efficiently engaged for deeper learning by reflection.^30^ Learning portfolios augment trainees’ long-loop learning by engaging supervisors or peers when challenged by complex patients,^12^ which was not evident in this study.

Compared with previous SA studies,^30^ workplace-based learning opportunities occurred during patient interactions, integrating feedback and FP engagements. Additional enablers in this study were peer learning, student training, and attending district activities such as CME and mortality and morbidity meetings. Learning occurred when exposed to patient loads based on community needs, experience with undifferentiated and holistic care and adequate hands-on practice, as found before.^59^ Resource challenges and a lack of district and university support^29^ also emerged as findings. Participation in district clinical activities was a major contributor to WBL, including community-oriented primary care, mortality and morbidity meetings and quality improvement projects. There were primary planned opportunities with FPs, but secondary opportunities occurred while engaging and immersing in the clinical workplace during patient interactions, as described in studies conducted in high-income countries.^60^ While FPs are expected to fulfil leadership and governance roles in SA,^6^ this study showed that registrars were not provided sufficient opportunities to learn or practice leadership and governance roles during training.

Self-directed learning behaviours and professionalism were variable among registrars, although FPs and registrars differed in their opinions. Trainees’ self-motivation^17^ and positive approach to feedback augmented self-directed learning.^12^ Self-directed learning and professional behaviours are essential registrar competencies and part of the national programmatic learning outcomes.^6^ Professionalism and self-directed learning are critical competencies to be attained by postgraduate FM trainees in many high-income countries (as prescribed in the Canadian competency FM framework^4,5^ and Accreditation Council for Graduate Medical Education [ACGME] Program Requirements for Graduate Medical Education in FM^3^). The availability of more explicit guidelines on programme requirements explaining how decentralised clinical training should be implemented would assist in a more standardised approach, as performed in high-income countries.^3,5^ More explicit guidelines on registrar professionalism, supervisor roles and characteristics, and supervisor–trainee relationships could be included.

A supportive learning environment requires good supervision opportunities and mutual observation, including modelling during practice, provision for narrative feedback, sufficient resources, additional organisational support, a manageable workload and time to reflect on patient interactions,^13,25^ which were found challenging in these settings. To optimise WBL, the learning environment should have a manageable workload and should address trainees’ well-being.^3,12,51^ The need for protected time is necessary, as work pressure impedes WBL.^26,27,51^ District management support is imperative for the growth of decentralised clinical training sites,^28^ which were also challenging, according to participants. Inadequate FP trainers and material resources, such as essential equipment and mannequins, negatively impacted WBL. Adequate material resources, including training space with adequate lighting, less background noise, comfortable seating arrangements and equipment availability, are all prerequisites for a conducive learning environment,^15,26,27,51^ the lack of which were found to be barriers.

The findings underscore the need for ongoing faculty development of supervisors focused on enhancing teaching and learning. Clinical educators typically have not undergone educational training in preparation for their supervisory roles and may thus have little understanding of relevant learning theories such as social learning theory^61,62^ or how to promote self-directed learning. There needs to be greater awareness around and more opportunities for FPs to attend training that influence WBL and supervision. The courses are offered by the SA Academy on areas such as postgraduate supervision, supervisory feedback, WBL and WPBAs. Participation in fellowship or masters’ programmes offered by the university could also improve clinical trainer skills as medical educators. Peer mentoring for personal and professional growth among trainers and trainees while working as a team in their workplace contexts should be encouraged. Faculty development should involve both ‘local faculty’ and ‘extended faculty’^63^ such as medical practitioners, nurses and multidisciplinary team members who play beneficial roles in registrar WBL enhancing interprofessional learning. For registrars, how to best utilise WBL opportunities by applying adult learning principles, feedback engagement and more reflective behaviours should be introduced and engaged earlier in their training.

### Study limitations

Exploring the WBL opportunities in decentralised sites of one university may have affected the transferability of the results to other similar contexts, although it is possible to some extent in case studies.^36^ Previous experience with the study contexts for the primary researcher may have influenced the data collection and analysis, but measures were taken to minimise this.

### Recommendations

Based on this study, we recommend maximising WBL opportunities and addressing contextual challenges for registrars. Peer learning and more supervisory engagement with mutual observation during trainees’ clinical practice need to occur. Adequate usage of the learning portfolio as a reflective tool, reflection on the supervisor’s feedback, and utilising interprofessional learning is encouraged. Participation in multiple FP roles and responsibilities, such as leadership and governance and staff and student training by registrars, can be more frequent. We recommend sufficient organisational support from district management and the university and improved resource availability to enhance WBL opportunities across individual sites. More explicit guidelines or policies on decentralised training programme implementation nationally could assist in optimising and even standardising WBL opportunities.

The in-depth understanding of WBL opportunities derived from this study will be integrated as a ‘process’ in the evaluation of the FM training programme using logic model. These findings, together, with those from other parts of the larger study (resource availability, postgraduate supervision and supervisory feedback)^32,33,34^ will be evaluated as the inputs, processes and outputs of the logic model. The overall logic model improved understanding of these factors and their relationships, which will be utilised for improving the programme as a whole.

## Conclusion

This study was an in-depth exploration of perceptions of postgraduate learning opportunities for FM training in the clinical workplace in SA. Self-directed learning, peer learning, student training and participation in district activities were identified as strengths. Interaction with supervisors, peers and other professionals could augment WBL opportunities. More reflection on supervisory feedback, registrar professionalism, learning portfolio utilisation and interprofessional learning is needed. Well-resourced facilities and exposure to various complex patients promote WBL, while excessive workloads, inadequate resources, and insufficient district management support impede learning. Strengthening the utilisation of available opportunities while addressing the challenges can maximise WBL in decentralised sites. Optimising learning opportunities in clinical environments provides superior learning experiences at sites, translating to better patient healthcare within communities. This study provides several areas for future research. Not only does it underscore the need for ongoing training programme evaluation, it creates possibilities for exploring the influence of respondents’ background on WBL and cross-case analyses of WBL across different training districts and sites, both nationally and internationally.
